# Nanog down-regulates the Wnt signaling pathway via β-catenin phosphorylation during epidermal stem cell proliferation and differentiation

**DOI:** 10.1186/2045-3701-5-5

**Published:** 2015-01-27

**Authors:** Peng Cheng, Xuying Sun, Delong Yin, Fei Xu, Kaixiang Yang, Liang Qin, Yonghui Dong, Fengjing Guo, Anmin Chen, Weikai Zhang, Hui Huang

**Affiliations:** Department of Orthopedics, Tongji Hospital, Tongji Medical College, Huazhong University of Science and Technology, Wuhan, 430030 P.R. China; Biological engineering and regenerative medicine center,Tongji Hospital, Tongji Medical College, Huazhong University of Science and Technology, Wuhan, 430030 P.R. China; Department of Orthopedics, The Third Hospital Affiliated to Guangzhou Medical University, Guangzhou, 510150 P.R. China

**Keywords:** Nanog, Wnt/β-catenin, Epidermal stem cells, Proliferation, Differentiation

## Abstract

**Background:**

Skin tissue homeostasis is maintained by a balance between the proliferation and differentiation of epidermal stem cells (EpSCs). EpSC proliferation and differentiation are complex processes regulated by many factors and signaling pathways. This study aimed to explore the connection between the Nanog and the Wnt/β-catenin pathway in the proliferation and differentiation of EpSCs.

**Results:**

Our results demonstrated that during the study period, EpSC underwent differentiation when incubated in the presence neuropeptide substance P (SP), there was an opposing expression trend of Nanog and β-catenin after SP treatment, which could be antagonized by the Wnt antagonist, Dkk-1. The transduced EpSCs had a greater proliferative ability than the SP treatment group and they did not undergo differentiation upon SP treatment. More important, β-catenin expression was down-regulated but phosphorylated β-catenin expression and phosphorylated GSK-3β expression was up-regulated upon Nanog overexpression.

**Conclusions:**

These results strongly suggest that Nanog plays an important role in maintaining the proliferation and differentiation homeostasis of EpSCs by promoting β-catenin phosphorylation via GSK-3β to inhibit the activity of the Wnt/β-catenin signaling pathway. This is important for precise regulation of proliferation and differentiation of EpSC in the application of tissue engineering.

## Background

Skin is the largest organ of the human body; it plays a key role in protecting the body against pathogens. The homeostasis of skin tissue is maintained by rare but pluripotent epidermal stem cells (EpSCs) and their progeny, transient amplifying (TA) cells [1, 2]. As pluripotent cells, EpSCs not only have an unlimited self-renewal capability to maintain a certain population but they also differentiate to form structures such as hair follicles and sebaceous glands [2, 3].

Transcription factors and signals determine whether EpSCs undergo self-renewal or differentiation. Several studies have established the central role of Nanog in maintaining pluripotency and preventing differentiation [4–7]. Nanog simultaneously increases the expression of genes conferring pluripotency and decreases the expression of genes driving cell differentiation. Increasing evidence also supports the involvement of the Wnt/β-catenin signaling pathway in the self-renewal and differentiation of EpSCs [8–10]. β-catenin is of central importance in the Wnt/β-catenin signaling pathway. Numerous articles have also reported that the fate of EpSC is controlled by an intricate relationship between different signaling pathways and transcription factors, especially Nanog and the Wnt/β-catenin signaling pathway [11–14]. However, previous research has not been able to clearly reveal the underlying molecular mechanism of EpSC proliferation and differentiation. Thus, the relationship between Nanog and the Wnt/β-catenin signaling pathway in EpSC proliferation and differentiation is of great interest.

In this study, we evaluated the relationship between the Wnt/β-catenin signaling pathway and Nanog in the proliferation and differentiation of EpSCs. Rat EpSCs were infected with lentivirus overexpressing Nanog. SP,a neuropeptide present in primary sensory neurons,belongs to the tachykinin family and can activate Wnt/β-catenin signaling pathway [15]. Transduced or untransduced cells were subsequently incubated in the presence or absence of SP. The effect of Nanog and SP on EpSC proliferation was tested *in vitro.* Subsequently, the expression of β-catenin and Nanog were evaluated in SP treated EpSCs using western blotting and PCR. In addition, the effect of Nanog on β-catenin expression was investigated in EpSCs using a lentivirus infection and/or SP treatment.

## Results

### Characterization of EpSCs

**Figure 1:**
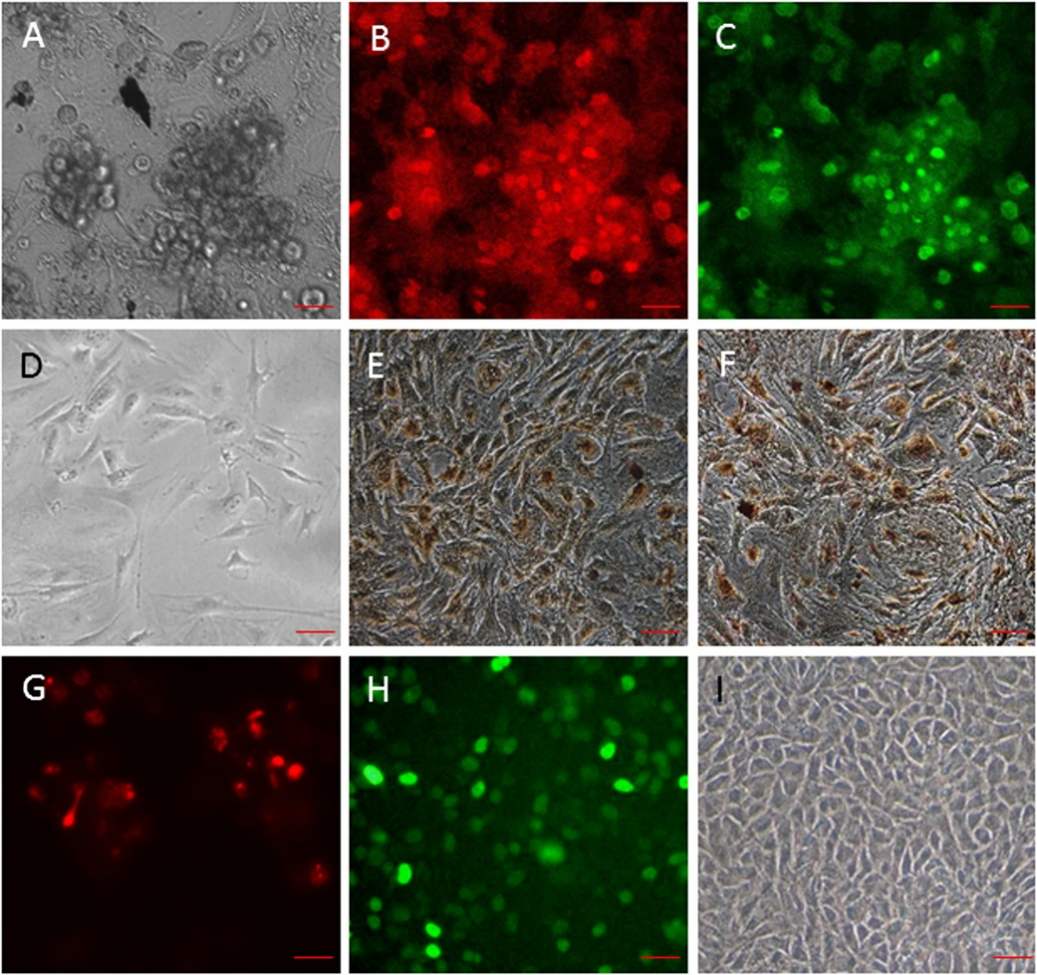
**Characterization of the isolated EpSCs.** The morphology of the isolated cells are bird nest-like or slabstone-like **(A)**. The isolated cells positively express CD34 **(B)** and β1 integrin **(C)** as detected by immunofluorescence. **(D)** EpSCs treated with 10^-7^ M for 12 days showed polygonal or long spindle morphology. **(E)** CK18 expression in these SP-induced EpSCs indicated these cells were differentiated, as demonstrated by immunocytochemistry. **(F)** Wnt agonist triggers EpSC differentiation, as demonstrated by CK18 immunocytochemistry. **(G)** CD34 positive expression in the SP with Dkk-1 treated EpSCs demonstrated that these cells were still stem cells, as detected by immunofluorescence. **(H)** Fluorescence microscopy image of the transduced EpSCs. **(I)** The transduced EpSCs did not undergo differentiation upon SP treatment. Scale bar =20 μm.

Rat EpSCs were isolated using rapid substrate attachment. Collagen type IV was used in this study to isolate EpSCs because collagen type IV is the ligand of β1 integrin, which is a potent cell marker of EpSCs. The rapidly adherent cells demonstrated stem cell characteristics as bird nest-like or slabstone-like (Figure 1A), which is the typical morphology of EpSCs. The expression of CD34 and β1 integrin was examined by immunofluorescence. Both CD34 (Figure 1B) and β1 integrin (Figure 1C) were highly expressed in the isolated cells, which proved that these isolated population were the EpSCs [16, 17]. On the other hand, EpSC differentiation was detected after 12 days of 10^-7^ M SP treatment or Wnt agonist treatment. Differentiated EpSCs showed the special morphologies as long spindle or polygonal (Figure 1D). Furthermore, differentiated EpSCs expressed CK18, which are expressed in the epithelial cells [18], as demonstrated by immunocytochemistry (Figures 1E, 1F). However, Wnt antagonist Dkk-1 can antagonize SP induced EpSC differentiation. CD34 positive expression in the SP with Dkk-1 treated EpSCs (Figure 1G) indicated that these cells were not differentiated. After infection with lentivirus-Nanog, EpSCs produced green fluorescence under a fluorescent microscope (Figure 1H). The transduced EpSCs did not undergo differentiation during the study period (Figure 1I).

### Proliferative ability of EpSCs infected with lentivirus or treated with SP *in vitro*

**Figure 2:**
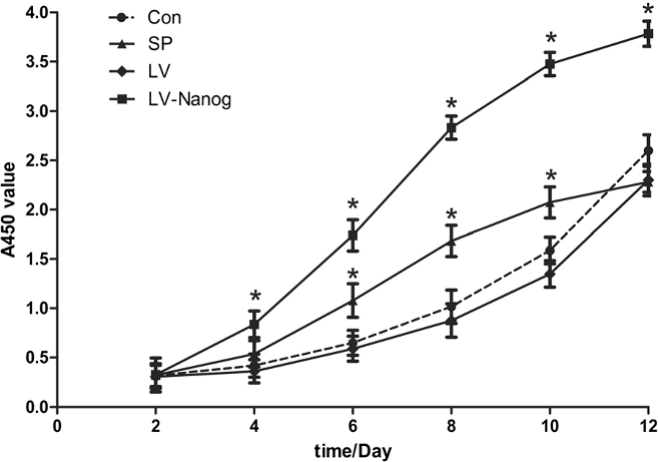
**Proliferative ability of EpSCs** ***in vitro*** **, as measured by the CCK-8 assay.**

A_450,_ which represents EpSC proliferative ability, was tested at one-day intervals from day 2 to day 12 using a CCK-8 kit. A strong linear increase in A_450_ between day 2 and day 12 was observed, indicating EpSC proliferation. The proliferative ability of EpSCs infected with control lentivirus vector was similar to the control, which had no significant difference at any time point. However, the proliferative ability of transduced EpSCs was much greater than the SP treatment group, control lentivirus vector infected group, and control during the study period. In addition, EpSCs treated with SP proliferated faster than the control in the first 10 days; on day 12, the proliferative rate of SP treated group reached a plateau while the control continued to rapidly proliferate (Figure 2) (P < 0.05).

### The expression of Nanog and β-catenin in EpSCs treated with SP

**Figure 3:**
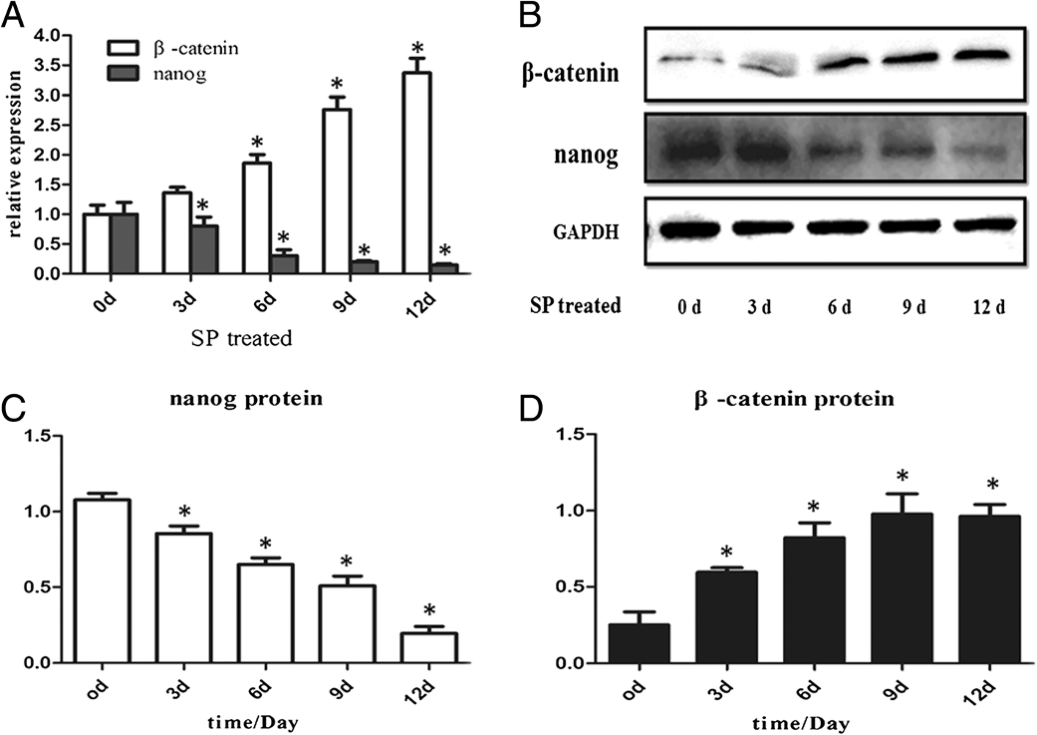
**Expression of β-catenin and Nanog in EpSCs treated with SP.** β-catenin and Nanog were measured at different time points (day 0-day 12) at the mRNA (q-PCR) and protein (western blot) level **(A and B)**. The bands of each protein were quantitatively analyzed **(C and D)**. Both q-PCR and western blot analysis showed that β-catenin expression was significantly higher on or after day 3 than at day 0. Nanog and β-catenin expression had an inverse relationship (*P < 0.05).

To reveal change in expression of Nanog and β-catenin in the EpSC differentiation process induced by 10^-7^ M SP, we detected Nanog and β-catenin expression at the mRNA and protein level every three days from day 0 to day 12. q-PCR analysis revealed that Nanog expression decreased. Nanog expression was significantly lower from days 3–12 than at day 0 (P < 0.05). However, β-catenin expression increased significantly from days 3–12 compared to day 0 (P < 0.05) (Figure 3A). Western blot results were similar to q-PCR results (Figures 3B, 3C, 3D).

**Figure 4:**
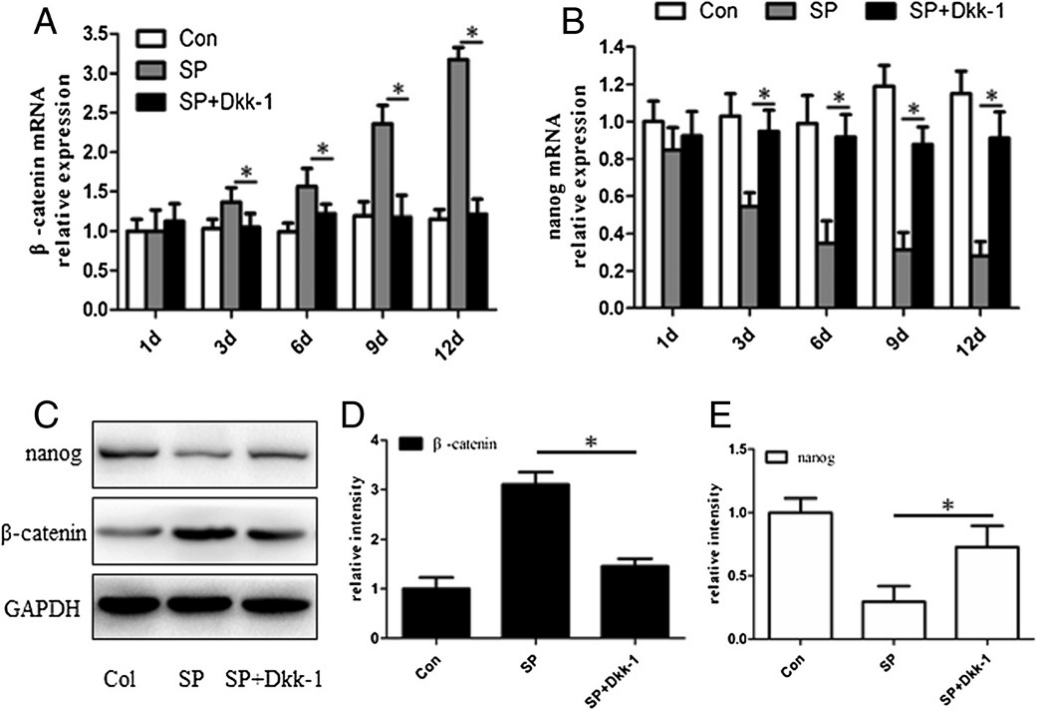
**Expression of β-catenin and Nanog in EpSCs treated with Wnt antagonist, Dkk-1.** Real-time PCR analysis of β-catenin mRNA **(A)** and Nanog mRNA **(B)** were obtained at different time points (day 1- day 12) in the control, SP treated group and SP with Dkk-1 treated group. The result showed that there were significant differences between the SP group and SP with Dkk-1 group on or after day 3 (*P < 0.05). At day 12, protein of Nanog and β-catenin were also detected **(C)**. The bands of both proteins in each group were analyzed quantitatively **(D and E)**. Werstern blot results were similar to q-PCR results (Con: control; SP: SP treated group; SP + Dkk-1: SP with Dkk-1 treated group).

To confirm that the expression of Nanog and β-catenin in the EpSC differentiation process induced by 10^-7^ M SP was related to Wnt/β-catenin signaling pathway, we used a specific Wnt antagonist, Dkk-1, and then detected Nanog and β-catenin expression at the mRNA and protein level every three days from day 0 to day 12. q-PCR analysis revealed that at any time on or after day 3 β-catenin expression was lower and Nanog expression was higher in both SP and Dkk-1treatment group than SP treatment group(P < 0.05) (Figure 4A and 4B). At day 12, protein of Nanog and β-catenin were also detected. The western blotting results were in accordance with q-PCR results. (P < 0.05) (Figures 4C, 4D and 4E).

### The expression of Nanog and β-catenin in EpSCs infected with lentivirus

**Figure 5:**
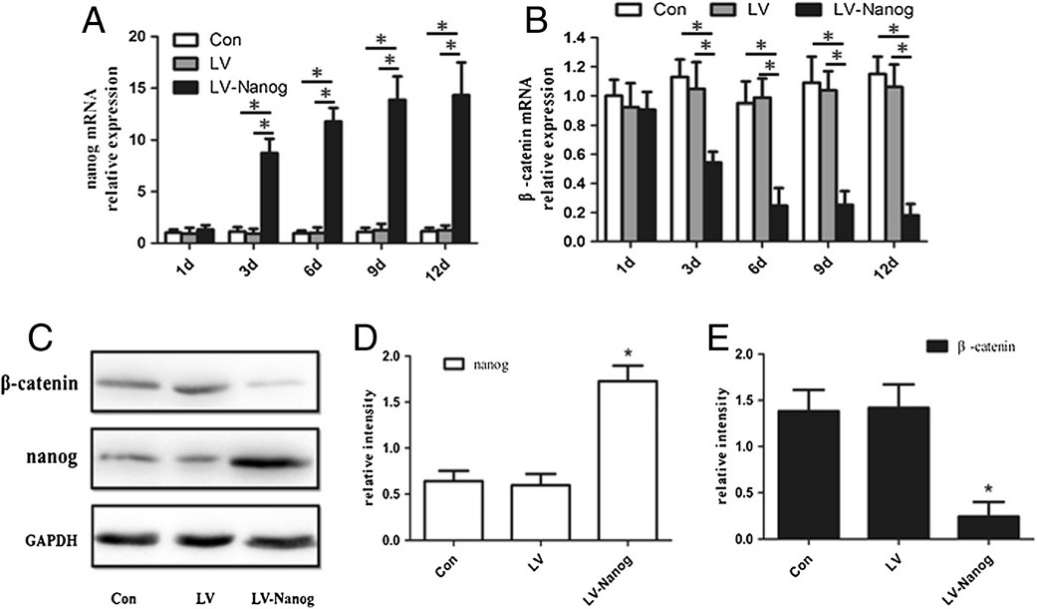
**Expression of β-catenin and Nanog in EpSCs infected with lentivirus.** Real-time PCR analysis of Nanog mRNA **(A)** and β-catenin mRNA **(B)** were obtained at different time points (day 1- day 12) in the control, control lentivirus vector infected group and transduced group. The results showed that there were no significant differences between the control and control lentivirus vector infected group. However, significant differences existed in the transduced group with the control on or after day 3(*P < 0.05). At day 12, protein of Nanog and β-catenin were also detected **(C)**. The bands of both proteins in each group were analyzed quantitatively **(D and E)**. Western blot results were similar to q-PCR results (Con: control; LV: control lentivirus vector infected group; LV-Nanog: transduced group).

To reveal the effect of Nanog overexpression on β-catenin and confirm the lentivirus itself do not affect the expression of Nanog or β-catenin, we infected EpSCs with lentivirus loaded with or without Nanog. Nanog and β-catenin expression at mRNA were detected at different time point (day 1- day 12) (Figures 5A and 5B). The results of q-PCR revealed that Nanog expression in the lentivirus-Nanog group increased remarkably at day 3, and continuely increased every day. However, β-catenin expression decreased from day 3, which were quite opposite to the Nanog expression. At day 12, protein of Nanog and β-catenin were also detected (Figures 5C, 5D and 5E). Western blot results were similar to q-PCR results. Besides, there were significant differences between the transduced group and control both at mRNA and protein level. And more, there was no significant difference between the control lentivirus vector infected group and control at either mRNA or protein level (Figure 5) (p < 0.05).

### Negative feedback of Nanog on the Wnt /β-catenin signaling pathway

**Figure 6:**
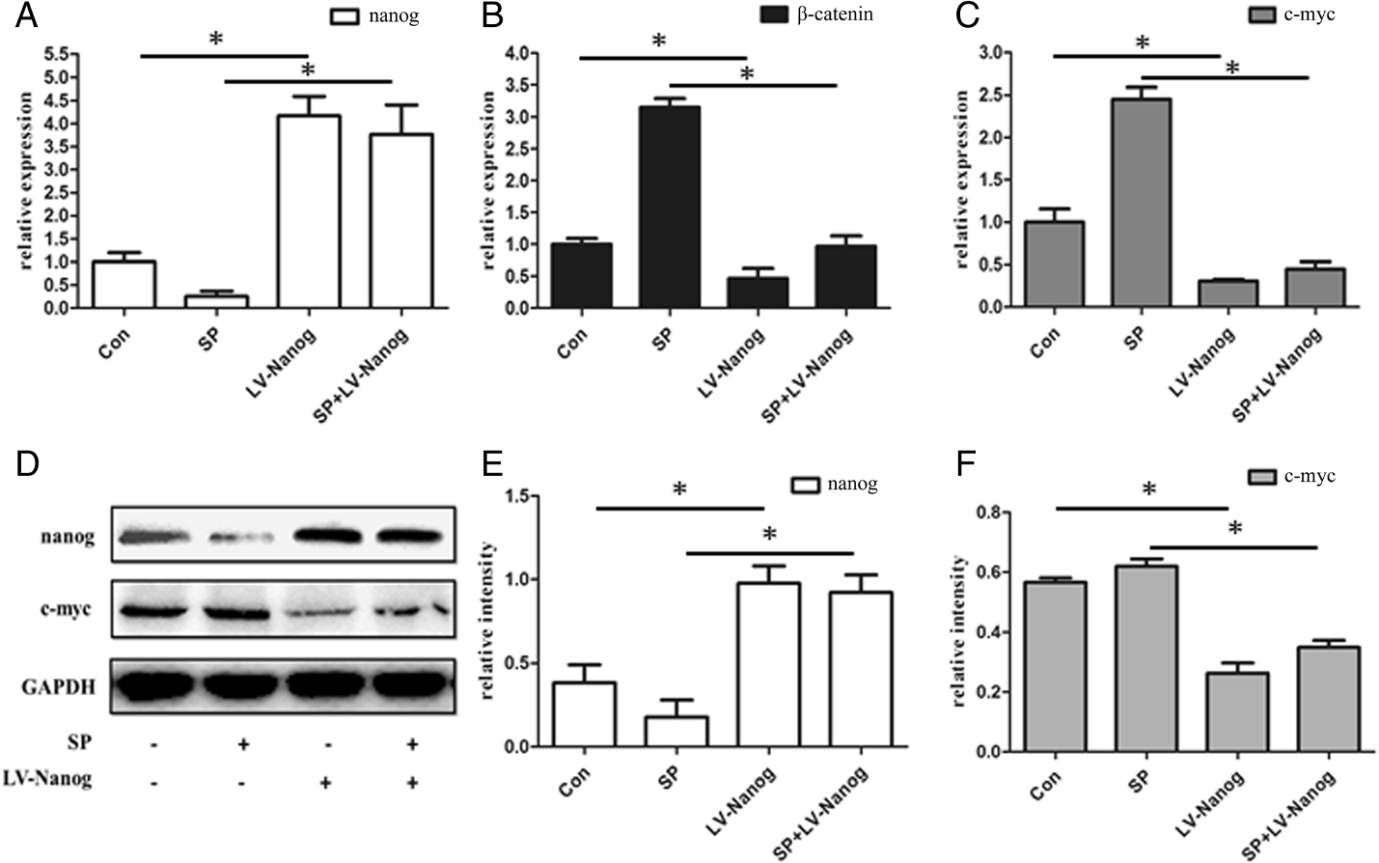
**Overexpression of Nanog inhibits the Wnt signaling pathway.** Real-time PCR analysis of gene expression (**(A)** Nanog, **(B)** β-catenin, and **(C)** c-myc) of the EpSCs in the control, SP-treated group, transduced group, and the combination treatment group. At the protein level, Nanog, c-myc, and total β-catenin were also detected **(D)**. The bands of Nanog and c-myc protein in each group were analyzed quantitatively **(E and F)**. (*P < 0.05) (Con: control; SP: substance P treated group; LV-Nanog: transduced group).

To explore how Nanog affects the Wnt/β-catenin signaling pathway, EpSCs infected with lentivirus encoding Nanog were incubated in the presence or absence of SP. Nanog, β-catenin and c-myc in Wnt/β-catenin signaling pathway were detected both at mRNA and protein level. At the mRNA level, Nanog expression in transduced cells in the absence or presence of SP was significantly higher than the control or SP-only treatment groups (Figure 6A) (P < 0.05). In contrast to Nanog expression, pt]?>β-catenin expression in transduced cells in the absence or presence of SP was significantly lower than the control or SP-only treatment groups (Figure 6B) (P < 0.05). C-myc, a downstream gene in the Wnt/β-catenin signaling pathway, trended with β-catenin expression (Figure 6C). The western blotting results were in accordance with q-PCR data. (Figures 5D, 5E, and 6 F).

**Figure 7:**
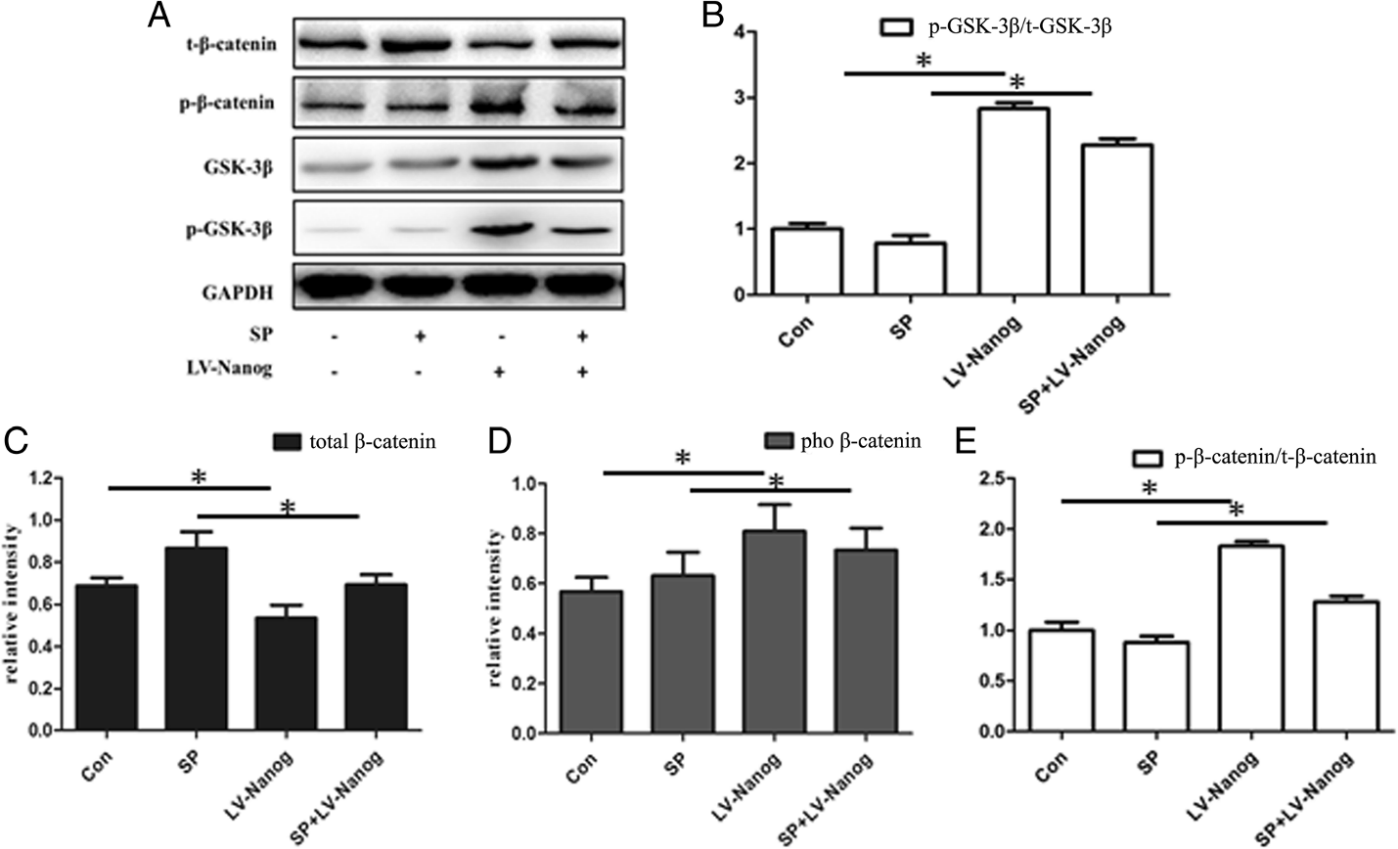
**Overexpression of Nanog promotes β-catenin phosphorylation.** The protein of total and phosphorylated GSK-3β,total and phosphorylated β-catenin were detected **(A)**. The ratio of phosphorylated GSK-3β to total GSK-3β was an indicator to inactivation of β-catenin **(B)**. The bands of β-catenin protein in each group were analyzed quantitatively **(C and D)**. The ratio of phosphorylated β-catenin to total β-catenin was regarded as the inactive β-catenin **(E)**. (*P < 0.05) (Con: control; SP: substance P treated group; LV-Nanog: transduced group).

To demonstrate the change in β-catenin expression in detail, total β-catenin,phosphorylated β-catenin, total GSK-3β protein and phosphorylated GSK-3β protein were also measured using western blotting (Figure 7A). Figure 7C shows that total β-catenin protein expression was significantly higher in the control and SP treated groups compared with transduced group in the absence or presence of SP (P < 0.05). However, phosphorylated β-catenin expression in EpSCs, which is inactivated β-catenin, was significantly different compared with total β-catenin protein expression (P < 0.05) (Figure 7D). Phosphorylated β-catenin protein expression in the control and SP treated groups was significantly lower than transduced cells in the absence or presence of SP. This result is the opposite of the results obtained with total β-catenin protein expression. Further analysis could be obtained, the ratio of phosphorylated β-catenin to total β-catenin was significantly high in transduced EpSCs (Figure 7E) (P < 0.05). In addition, the ratio of phosphorylated GSK-3β to total GSK-3β was another indicator of inactivated β-catenin. With the intensity quantitative analysis, it had a similar trendency with the ratio of phosphorylated β-catenin to total β-catenin (Figure 7B) (P < 0.05).

## Discussion

As the primary barrier in the human body, skin protects humans from infection, radiation and wounds. However, skin, especially the epidermis, may be injured by external insults. The proliferation and differentiation of EpSCs and their progeny of TA cells greatly affect skin repair [2, 19, 20]. This process is controlled by an intricate relationship between different signaling pathways and transcription factors, especially the Wnt/β-catenin signaling pathway and Nanog [21–23].

Nanog is a pluripotent state-specific transcription factor that plays a critical role in regulating cell fate during embryonic development, maintaining pluripotent epiblasts and preventing differentiation [24, 25]. Nanog is also proposed as a transcription repressor that inhibits important genes for cell differentiation [7, 26]. Our results showed that the proliferation ability of transduced EpSCs is much greater than the control and SP treated groups (Figure 2). Additionally, transduced EpSCs treated with SP did not undergo differentiation (Figure 1I). These results using EpSCs agree with previous reports that Nanog can promote proliferation and inhibit differentiation of stem cells. On the other hand, accumulating evidence indicates that the Wnt/β-catenin signaling pathway plays a critical role in the lineage decision/commitment process [10, 23, 27, 28]. In contrast, there are also some reports that the Wnt/β-catenin signaling pathway plays a pivotal role in the maintenance of pluripotency as well as in the process of somatic cell reprogramming [9, 29–31]. These dramatically different observations upon activation of the Wnt signaling cascade have fueled enormous controversy concerning the role of Wnt signaling in the maintenance of differentiation potency and the induction of stem cells. Our results in EpSCs support the former conclusion. In this study, EpSCs were differentiated when they were treated with a Wnt agonist (Figure 1F) and the differentiation was antagonized by Dkk-1(Figure 1G). This result supports evidence that the Wnt/β-catenin signal pathway affects EpSCs differentiation.

It is noteworthy that treatment with 10^-7^ M SP caused a rapid proliferation of EpSCs into TA cells in the first twelve days, which subsequently had a limited proliferative ability (Figure 2). This result agrees with our previous study regarding the effect of SP on EpSCs [32]. Previous reports also support our findings [33, 34]. From day 12, the EpSCs underwent differentiation, manifesting the typical morphology and expressing CK18, which is an EpSCs differential marker [35, 36] (Figures 1D, 1E). This is a new observation compared to our previous research. In this process, the expression of the pluripotency protein Nanog decreased significantly. However, β-catenin transcription and translation increased (Figure 3). Besides, the increase of β-catenin could be counteracted by Dkk-1 (Figure 4). The opposing expression trends of Nanog and β-catenin suggested that there should be an interaction between them (Figures 3 and 4).

To prove this hypothesis, we infected lentivirus-Nanog into EpSCs *in vitro* (Figure 1H). CCK-8 results revealed that the transduced EpSCs had a strong proliferative ability, while the control lentivirus vector infection showed no effect on the EpSCs proliferative ability (Figure 2). Besides, the control lentivirus vector infection also did not interfere with Nanog and β-catenin expression either (Figure 5). Nanog counteracted SP-induced cell differentiation (Figures 1I and 6A). The most important result is that β-catenin expression in the transduced EpSCs was notably decreased as measured by q-PCR and western blot (Figure 6). This result suggests that Nanog can inhibit EpSC differentiation by inhibiting β-catenin expression. C-myc, a downstream target of Wnt/β-catenin signaling pathway, binds to promoter sequences after activation by β-catenin-TCF complexes. Haegele et al. found that the activation of c-myc and its downstream targets Cyclin-dependent kinase 2 (CDK) and Cyclin A could up-regulate BMPs and cause embryonic stem cell differentiation [31]. In the SP-treated groups, c-myc expression of the transduced EpSCs was also suppressed both at mRNA and protein level (Figures 6C, 6F). The change in c-myc expression agreed with β-catenin changes in the transduced EpSCs, which indirectly demonstrates that β-catenin is involved in EpSC differentiation. This result further proved that Nanog can maintain EpSC pluripotency and prevent differentiation by inhibiting the β-catenin expression.

To explore the potential mechanism of this process, we measured the levels of phosphorylated β-catenin protein, phosphorylated GSK-3β and total GSK-3β in each group. It is well known that the activity of the Wnt/β-catenin signaling pathway is affected by glycogen synthase kinase (GSK). GSK-3β is phosphorylated and results in β-catenin phosphorylation and its subsequent degradation by the ubiquitin-mediated pathway [29, 37]. In this experiment, we found that the ratio of phosphorylated β-catenin to total β-catenin paralleled Nanog expression in each group (Figure 7E), which is in common with the ratio of phosphorylated GSK-3β to total GSK-3β (Figure 7B). Therefore, we conclude that Nanog inhibits β-catenin activity through the phosphorylation of β-catenin via GSK-3β. Thus, we can infer the underlying process by which EpSCs execute their function. When skin is not injured, Nanog maintains the multi-potential and proliferative state of EpSCs through inhibiting β-catenin expression via β-catenin phosphorylation. However, when skin needs to repair an injury, negative feedback from β-catenin on Nanog suppresses its expression and induces EpSC differentiation. The whole process balances the differentiation and proliferation of EpSCs.

## Conclusion

In summary, our study showed that Nanog and the Wnt/β-catenin signaling pathway are involved in the regulation of EpSC proliferation and differentiation, which is necessary to maintain EpSCs homeostasis in the skin. The underlying mechanism is that Nanog inhibits the activity of the Wnt/β-catenin signaling pathway via GSK-3β mediated β-catenin phosphorylation. This finding represents an important step towards understanding the precise regulation of EpSC proliferation and differentiation, which has important applications in tissue engineering.

## Materials and methods

### Cell culture

Sprague–Dawley (SD) male rats were purchased from HUST Laboratory Animal Center (Wuhan, China). The Ethics Committee of Tongji Medical College, Huazhong University of Science and Technology, approved all animal care and experimental procedures. The EpSCs isolated procedure was regarded as a standard protocol which was first repoted by Liu Y [16]. Skin tissue was obtained from the back of neonatal SD rats by plastic surgical procedures and was washed in phosphate-buffered solution (PBS). Connective tissue and subcutaneous fat were removed. The skin sample was sterilized with 70% ethanol for 5 min, rinsed in PBS, and minced into 5-mm wide strips using a sharp scalpel; the strips were treated with 0.25% Dispase (Roche, Switzerland) solution at 4°C overnight. The epidermis was mechanically separated from the dermis and incubated in 0.25% trypsin solution at 37°C for 15 min to dissociate the cells; enzyme activity was then blocked with Dulbecco’s modified Eagle’s medium (DMEM; Gibco, USA) containing 10% fetal bovine serum (FBS; Gibco, USA), and the cells were suspended with a pipette. To remove any remaining tissue pieces, the cell suspension was filtered through a stainless steel mesh attached to a 60-mm cell culture plate. The cells were transferred to a 15-ml centrifuge tube and collected by centrifugation for 5 min at 250 *g*. To select stem cells, 1x10^6^ dissociated epidermal cells were plated onto collagen type IV (Sigma, USA) (100 μg/ml)-coated dishes at room temperature for 15 min. The unattached cells were removed, and the rapidly adherent epidermal cells were cultured with keratinocyte serum-free medium (K-SFM; Gibco, USA) supplemented with epidermal growth factor (EGF) (K-SFM; Gibco, USA), bovine pituitary extract (BPE; Gibco, USA), and 0.05 mM calcium chloride (CaCl_2_, Sigma, USA) at 37°C under 5% CO_2_ in a humidified incubator for two days before replacing the medium. The medium was changed every other day.

### Lentivirus vector construction and EpSC infection

The coding sequence of rat Nanog was PCR amplified from GV208: rat-Nanog using primers with AgeI/AgeI overhangs and was cloned into pTZ58 (Fermentas, Vilnus, Lithuania). The AgeI/AgeI fragment was then sub-cloned into pUbi (AgeI/AgeI) and pEGFP-C1 (Clontech, Mountain View, CA) (AgeI/AgeI) to generate Ubi-Nanog-EGFP encoding plasmids, respectively. To produce lentivirus, the pBABE-puro plasmids were co-infected along with the helper plasmids into 293 T cells, and the medium was harvested 36 h and 72 h after infection. EpSC infection was performed by incubating the cells in virus-enriched medium for 12 h, which included 4 μg/ml polybrene. Transduced cells were identified for EGFP expression under a fluorescence microscope. The transduced EpSCs were divided into two groups: the first group of EpSCs was treated with SP for 12 days after infection; the second group of EpSCs was cultured for 12 days. EpSCs were also infected with control lentivirus vector and cultured for 12 days. EpSCs without any treatment were used as control.

### Immunofluorescence

The isolated cells were cultured in a 6-well plate (10^4^ cells/well) for 24 h. The cells were rinsed five times with PBS, fixed with 4% paraformaldehyde solution for 15 min at room temperature, and permeated with 0.5% Triton X-100-PBS solution for 5 min. After blocking with 3% BSA in PBS for 30 min, the cells were respectively incubated with primary antibodies to anti-CD 34 antibody (rabbit polyclonal antibody, 1:50, Santa Cruz Biotechnology, Inc.) and anti-β1 integrin antibody (rabbit polyclonal antibody, 1:50, Boster Biological Technology, Ltd.) at 4°C overnight. The primary antibody binding was detected via the corresponding goat anti-rabbit IgG: Cy3 and goat anti-mouse IgG FITC (Boster Biological Technology, Ltd.). Staining was examined under a fluorescence microscope.

### Immunocytochemistry

The isolated cells were cultured in a 6-well plate (10^4^ cells/well) for 24 h. The cells were treated with 10^-7^ M SP or LiCl, a Wnt agonist, to induce differentiation for 12 days. The cells were rinsed five times with PBS, fixed with 4% paraformaldehyde solution for 15 min at room temperature, and permeated with 0.5% Triton X-100-PBS solution for 5 min. The cells were incubated with primary antibodies to anti-cytokeratin 18 antibody (CK18) (rabbit polyclonal antibody, 1:50, Santa Cruz Biotechnology, Inc.) at 4°C overnight. Subsequent procedures were performed according to the SP-9000 Histostain-Plus Kit instructions (ZSGB-BIO, Beijing, China). The cells were observed under a microscope (Bio-Rad, Hercules, CA, USA).

### Cell Counting Kit-8 (CCK-8) assay

EpSCs were plated in 96-well plates (2 × 10^4^ cells/well) and treated with collagen type IV. The cells were then divided into four groups: the first group was composed of cells without any treatment, the second group was composed of cells infected with control lentivirus vector, the third group was composed of transduced cells, and the forth group was composed of cells that were exposed to 10^-7^ M SP. Every other day after day 2 until day 12 post-treatment, 10 μL of CCK-8 (Boster, China) solutions were added to each well, and the samples were incubated for 2 h. The specific absorbance at 450 nm (A_450_) was determined using a Micro-plate Reader (Thermo Fisher, USA). All of the experiments were repeated independently at least three times, and each measurement was the average of 5 duplicate wells.

### Western blotting

The total cell lysates of EpSCs that underwent the proliferation and differentiation were obtained by lysing the cells in RIPA buffer (Boster, China) containing 50 mM Tris–HCl, 150 mM NaCl, 1% NP-40, 0.1% SDS, 0.5% sodium deoxycholate, 2 mM sodium fluoride, 1 mM EDTA, 1 mM EGTA and a protease inhibitor cocktail. The protein concentration was determined using the bicinchoninic acid protein assay (Beyotime, China). The proteins were separated by SDS-PAGE, transferred to nitrocellulose, blocked with bovine serum albumin and incubated with the following primary antibodies: rat anti-β-catenin (CST, USA) diluted 1:1000, rat anti-p-β-catenin (CST, USA) diluted 1:1000, rat anti-c-myc (CST, USA) diluted 1:1000, rat anti-Nanog (CST, USA) diluted 1:2000, rat anti-GSK3β (CST, USA) diluted 1:1000, rat anti-p-GSK3β (CST, USA) diluted 1:1000 or rat anti-GAPDH (Beijing Biosynthesis Biotechnology, China) diluted 1:1000 that was used as a loading control. The membrane was washed and incubated with the respective secondary antibodies conjugated with peroxidase. Chemiluminescence (Bio-Rad, USA) was used to detect protein.

### Real-time quantitative PCR

Total RNA was extracted using the TRIzol reagent (Invitrogen, USA), according to the manufacturer’s protocol. Using a spectrophotometer (Eppendorf, Germany), the RNA concentration and purity were determined by A_260_ and A_260_/A_280_ ratios, respectively. Total RNA was used to synthesize cDNA with a Super-Script II cDNA synthesis kit (Invitrogen Life Technologies, USA). The related genes of the Wnt/β-catenin signaling pathway and Nanog were assessed by quantitative real-time PCR using a SYBR-Green Master mix (Fermentas, Vilnius, Lithuania). Glyceraldehyde phosphate dehydrogenase (GAPDH) was selected as an internal control. The primer sequences are: Nanog (accession no. NM_001100781) forward, CCGTTGGGCTGACATGAGCGT and reverse, GGCAGGCATCGGCGAGGAAT; β-catenin (accession no. NM_053357) forward, AGCCCGTTGTACCGCTGGGA and reverse, CGCTGGGATGCCGCCAGATT; c-myc (accession no. NM_012603) forward, CATCATCCAGGACTGTATGTG and reverse, TGGAATCGGACGAGGTA; GAPDH (accession no. NM_017008) forward, TATGACTCTACCCACGGCAAGT and reverse, ATACTCAGCACCAGCATCACC.

### Statistical analysis

All experiments were repeated independently at least three times. The data are presented as the mean ± standard deviation (SD). The results were analyzed using SPSS 18.0 statistical software. Statistically significant differences were identified using Student’s t-test and an analysis of variance (ANOVA). The significance level was set to P < 0.05.
